# The Dynamic EEG Microstates in Mental Rotation

**DOI:** 10.3390/s18092920

**Published:** 2018-09-03

**Authors:** Wanzeng Kong, Luyun Wang, Jianhai Zhang, Qibin Zhao, Junfeng Sun

**Affiliations:** 1 School of Computer and Technology, Hangzhou Dianzi University, Hangzhou 310000, China; wangluyun@hdu.edu.cn (L.W.); jhzhang@hdu.edu.cn (J.Z.); 2 Fujian Key Laboratory of Rehabilitation Technology, Fuzhou 350000, China; 3 Tensor Learning Unit, RIKEN AIP, Tokyo 103-0027, Japan; qibin.zhao@riken.jp; 4 School of Biomedical Engineering, Shanghai Jiao Tong University, Shanghai 200000, China

**Keywords:** mental rotation, microstate, event-related potential, cognitive process, spatial-temporal, dynamic EEG

## Abstract

Mental rotation is generally analyzed based on event-related potential (ERP) in a time domain with several characteristic electrodes, but neglects the whole spatial-temporal brain pattern in the cognitive process which may reflect the underlying cognitive mechanism. In this paper, we mainly proposed an approach based on microstates to examine the encoding of mental rotation from the spatial-temporal changes of EEG signals. In particular, we collected EEG data from 11 healthy subjects in a mental rotation cognitive task using 12 different stimulus pictures representing left and right hands at various rotational angles. We applied the microstate method to investigate the microstates conveyed by the event-related potential extracted from EEG data during mental rotation, and obtained four microstate modes (referred to as modes A, B, C, D, respectively). Subsequently, we defined several measures, including microstate sequences, topographical map, hemispheric lateralization, and duration of microstate, to characterize the dynamics of microstates during mental rotation. We observed that (1) the microstates sequence had a specified progressing mode, i.e., A→B→A; (2) the activation of the right parietal occipital region was stronger than that of the left parietal occipital region according to the hemispheric lateralization of the microstates mode A; and (3) the duration of the second microstates mode A showed the shorter duration in the vertical stimuli, named “angle effect”.

## 1. Introduction

Mental rotation (MR) is a special visuo-spatial cognitive process of imagining and identifying a two- or three-dimensional object that is rotated away from its original upright position, which can reflect the ability of both the actual movement and the spatial cognition [1,2]. Generally, the cognitive process of mental rotation includes three stages: encoding of visual stimulation (0–300 ms), stage of mental rotation (300–700 ms) and decision response (700–1000 ms) [3]. Studies on both the underlying neural mechanism and clinical application of MR have been reported. For instance, Chen et al. studied the physiological mechanism of mental rotation for the depression patients, and demonstrated that the brain topographical map could be used as a clinical diagnostic indicator [4]. Yan et al. investigated the cognitive neural mechanism of mental rotation in stroke patients, and proposed the prospective applications of mental rotation in stroke rehabilitation [5].

A range of studies have demonstrated that event-related potential (ERP) is one powerful tool to investigate the mental rotation process [6,7,8,9]. Currently, the research on mental rotation are often carried out with the combination of ERP and behavioral data. ERP is an electrical brain response extracted from time-locked electroencephalography (EEG) signals by averaging across trials of the same type [10,11]. Heil et al. found a significant P300 component (a typical ERP component appears in about 300–500 ms after stimulus onset) in the parietal lobe in mental rotation task and thus proposed that the P300 component was a specific component to process spatial information [3]. Heath et al. examined the concurrent behavioral, ERP characteristics of a standard reaching task, and visuomotor mental rotation (VMR) tasks of $35^{\circ }$, $70^{\circ }$, and $105^{\circ }$ of rotation. They found that the P300 amplitude was larger for the standard compared to each VMR task [9]. However, the current mental rotation research of ERP generally examines the temporal changes of ERP in some specific channels, or examines the changes in the brain topographical maps of a particular component over time, while rarely examining from the perspective of the whole brain pattern over space. Generally, the brain performs a high cognitive function through the integration of multiple brain regions [12]. Therefore, investigation on the mental rotation from the spatial-temporal dynamics of ERP will improve our understanding of the mental rotation.

Functional microstate is defined as the quasi-stable configuration of multichannel EEG as being composed of a time sequence of nonoverlapping epochs [13,14]. Microstate analysis is an analytical method that can describe the spatial-temporal dynamics of brain activities. In particular, this method classifies each instantaneous multichannel evoked potential to some microstates based on classical K-means clustering [13,15,16,17,18,19,20,21,22,23,24,25,26,27,28]. For instance, Hu et al. showed the functional microstates of laser-evoked potentials (LEPs) elicited by the stimulation of the left hand, right hand, left foot and right foot. Four function microstates were commonly observed in all experimental conditions, located at the time intervals corresponding to the N1, N2, P2 and P4 components [29]. Von Wegner et al. presented an information-theoretical analysis of temporal dependencies in EEG microstate sequences during wakeful rest, and interpreted microstate sequences as discrete stochastic processes where each state corresponded to a representative scalp potential topography [30]. Dering et al. used a data-driven approach based on functional microstates by EEG data of viewing a physically matched face and butterfly images of participants, and they showed that the locus of endogenous effects also found with ERPs occurs in an N1 time range [31].These previous studies have shown that microstate analysis can be used to study spatial-temporal dynamics of brain and provide new insights for mental rotation.

With the observations above, we used the microstate analysis to investigate the spatial-temporal dynamics of the brain state during mental rotation tasks. Subsequently, we clustered the multichannel ERPs extracted from EEG signals into four microstate modes (A, B, C, D). We obtained the specified microstate sequence and assessed the relationship between the microstate sequence and ERP. Moreover, we observed the hemispheric lateralization in microstates mode A, and the significant “angle effect” in duration. The remainder of this paper was organized as follows. Section 2 represented subjects, experimental paradigm, EEG recording, EEG data preprocessing, and the methods of microstate analysis. Section 3 showed the experimental results. Consequently, Section 4 presents the discussion and then Section 5 presents the conclusions.

## 2. Materials and Methods

### 2.1. Experimental Paradigm

Eleven volunteers (mean age: 60.1 ± 6.9 years, range: 45–68 years, male/female: 6/5), who reported no history of seizures, neurological diseases, or psychiatric disorders, were recruited from the local community. All subjects were right-handed with normal or correct-to-normal vision and given the written informed consent.

In the experiment, left- or right-hand pictures were used as stimulus (9 cm × 9 cm) and randomly presented on the display with a viewing angle of approximately $2.5^{\circ }$ in height. Both left- and right-hand visual stimuli had six different directions: $0^{\circ }$, $60^{\circ }$, $120^{\circ }$, $180^{\circ }$, $240^{\circ }$, $300^{\circ }$. In total, there were 12 stimulus types, named as S1, S2, ..., S11, and S12, respectively, with the probabilities listed in Table 1. In the experiment, six blocks of visual stimuli were presented for subjects with 3–5 min inter-block break. Each block included 96 stimuli (48 left-hand stimuli and 48 right stimuli). E-prime (Psychology Software Tool Inc., Pittsburgh, PA, USA) was used to control the stimulation timing of visual stimuli and synchronization between behavioral and EEG data acquisition. In each trial, a visual stimulus picture was presented until the subject completed the judgment and pressed the response button. The inter-stimulus interval (ISI) of the visual picture stimulation was 800 ms. During the interval, a black cross symbol “+” was presented on the display. During the experiment, subjects were requested to pay their attention to the stimuli and keep minimal movements of head and eye. In addition, they were requested to press the left button (‘A’ key of the keyboard) for left-hand stimuli by the left index finger and the right button (‘L’ key of the keyboard) for right-hand stimuli by the right index finger. More details of the experiment can be found in [32]. This study was approved by the Ethics Committee of Shanghai Jiao Tong University and the Fifth People’s Hospital of Shanghai.

EEG signal was recorded using 32 channels Ag-AgCl electrodes by Vision Recorder (Version 1.03, Brain Products GmbH, Munich, Germany) and amplified and digitized at a sampling rate of 1000 Hz and impedance below 5 kΩ at each electrode.

### 2.2. EEG Data Preprocess

EEG signal was band-pass filtered into the range of 0.01–30 Hz, and eye-movement artifacts were removed with the recorded continuous horizontal and vertical electro-oculograms (EOGs) by means independent component analysis (ICA) method [33,34,35,36]. Furthermore, EEG signal was re-referenced to a common average reference. After re-reference and EOG removal steps, in total, there were 28 channels EEG signal in this study. Then, the EEG trials with artifacts greater than 100 μv or less than −100 μv were excluded. Finally, EEG data was segmented into trails of 1200 ms (from 200 ms pre-stimulus to 1000 ms post-stimulus) and the baseline of each trial was corrected using the pre-stimulus interval. All trails in the same stimulus type were averaged for an EEG analysis.

### 2.3. Methods

#### 2.3.1. The ICA Algorithm

The ICA is a statistical method that can separate mixed signals, including EEG signals, to maximize the separation of components in a statistically independent measure [36]. Generally, the ICA problem can be described as: $Y={\left[y_{1},y_{2},\cdots ,y_{N_{s}}\right]}^{T}$ be a vector of random observation of $N_{s}$ dimensions, and $S=[s_{1},s_{2},\cdots ,s_{M}]$ is the original unobserved sources of *M*. The ICA mode can be expressed as:

(1) Y=AS,

where *A* is a full-rank scalar matrix as $\left[N_{s}\times M\right]$ that mixes ICs back to observed signals, $N_{s}$ is the number of the channels to record EEG data, *M* is the number of the original unobserved sources. Because we do not know the effective number of independent signals, we suppose that the number of source signals is equal to the number of observation signals, that is, N=M. Given the EEG data, the ICA algorithm can calculate the mixed matrix *A* and the independent sources of $N_{s}$. The matrix *A* is expressed as below:

(2) $$A=\left(\begin{array}{cccc} a_{11} & a_{12} & \cdots & a_{1N_{s}},\\ a_{21} & a_{22} & \cdots & a_{2N_{s}},\\ \cdots & \cdots & & \cdots \\ a_{N1} & a_{N2} & \cdots & a_{N_{s}N_{s}} \end{array}\right),$$

where $a_{ij}\left(i\leq i,j\leq N_{s}\right)$ is the transfer coefficient from the *j*-th source to the *i*-th observed channel signal. In addition, the aim of ICA is to find out the linear unmixing matrix *W* and acquire the ICs under the conditions of independent criterions, which is an inverse problem of Equation (1), so that:

(3) S=WY.

**Figure 1:**
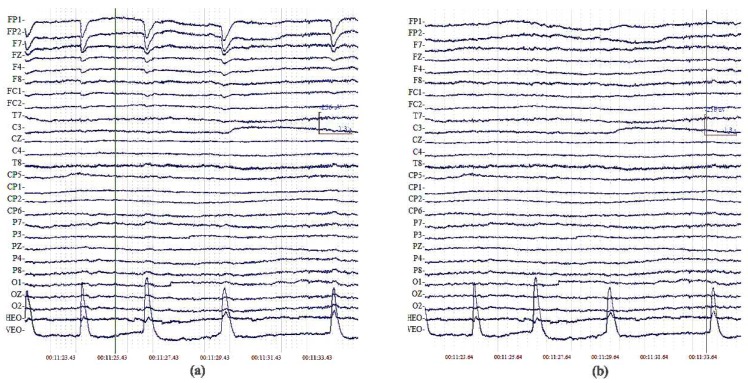
(**a**) original signals; (**b**) reconstructed signals after blink artifact removal by the ICA method.

Therefore, the ICA algorithm can effectively separate the eye-movement artifacts from weaker EEG signals. Let us take a look at the original signals and the reconstructed signals of one subject after blink artifact removal by the ICA method (Figure 1).

#### 2.3.2. The Microstates Model

In this work, we collected the EEG data from the 11 subjects in 28 channels. We preprocessed the original EEG data to 11 subjects in 12 different visual stimuli and then got the EEG data *X* of ERP by averaging all the trails in the same stimulus type of each subject. The EEG data $X=[V_{1},V_{2},\cdots ,V_{t},\cdots ,V_{N_{T}}]$ is a $N_{s}\times N_{T}$ dimensional matrix, where $V_{t}={\left[v_{1}^{t},v_{2}^{t},\cdots ,v_{N_{s}}^{t}\right]}^{T}$ is a $N_{s}\times 1$ vector consisting of the scalp electric potential measurements at time instant $t\;(t=1,\cdots ,N_{T})$, $N_{s}$ is the number of the channels to record EEG data, and $N_{T}$ is the number of sampling points.

Each time point *t* of the brain electrical activity belongs to a kind of the microstate Γ. Therefore, scalp EEG activity in a time period can be expressed by a set of normalized vectors Γ.

The microstate model for average reference data can be expressed as

(4) $$V_{t}=\sum_{k=1}^{N_{\mu }}a_{kt}\Gamma_{k},$$

where $\Gamma_{k}$ is the normalized $N_{s}\times 1$ vector representing the *k*-th microstate, $a_{kt}$ is the *k*-th microstate intensity at time instant *t*, and $N_{\mu }$ is the number of the different microstates.

In order to allow for nonoverlapping microstates at each time instant *t*, all $a_{kt}$ must be zero except for one. Therefore, at each time instant, the summation in Equation (4) reduces to a single nonzero term, corresponding to a single active microstate. Formally, the following constraints must be satisfied:

(5) $$\left.\begin{array}{cc} a_{k_{1}t}\cdot a_{k_{2}t}= & 0,\forall k_{1}\neq k_{2},\forall t,\\ \sum_{k=1}^{N_{\mu }}a_{kt}^{2}\geq & 0,\forall t. \end{array}\right\}$$

For given $N_{\mu }$, the model parameters can be estimated by minimizing the function

(6) $$F=\frac{1}{N_{T}\left(N_{s}-1\right)}\sum_{t=1}^{N_{T}}{∥V_{t}-\sum_{k=1}^{N\mu }a_{kt}\Gamma_{k}∥}^{2}$$

with respect to all $\Gamma_{k}$ and $a_{kt}$, under constraints Equation (5). In Equation (6), ∥·∥ denotes the norm of the vector.

The minimizing problem of Equation (6) could be solved by the grouped variable coordinate descent method [37], which consists of iterations alternations between two basic steps. In the first step, the normalized and linearly independent set of vectors $\Gamma_{k}\;\left(k=1,\cdots ,N_{\mu }\right)$ is given. The minimization of Equation (6) is obtained as follows with respect to $a_{kt}$ under the constraint Equation (5). The orthogonal squared distance between each measurement vector and each microstate is computed

(7) $$d_{kt}^{2}=V_{t}^{\prime }\cdot V_{t}-{\left(V_{t}^{\prime }\cdot \Gamma_{k}\right)}^{2}.$$

Each measurement is labeled as belonging to the microstate to which it is closest to. Let $L_{t}$ denote the microstate label, and then

(8) $${\hat{L}}_{t}=arg\underset{k}{min}\left\{d_{kt}^{2}\right\}.$$

The estimator for the nonzero $a_{kt}$, where $k={\hat{L}}_{t}$, is given by

(9) $$a_{kt}=V_{t}^{\prime }\cdot \Gamma_{k}.$$

This part of the algorithm consisting of Equations (7)–(9) is carried out for all vectors $V_{t}$.

In the second step, the labels $L_{t}$ are given, and the minimum of Equation (7) with respect to $\Gamma_{k}$ under constraint Equation (5) is obtained directly as the normalized eigenvector corresponding to the largest eigenvalue of the matrix

(10) $$S_{k}=\sum_{t|L_{t}=k}V_{t}\cdot V_{t}^{\prime }.$$

Note that the summation includes only time points for which $L_{t}=k$. Thus, $L_{t}$ corresponds to the microstate at each time instant *t*. Therefore,

(11) $$\Gamma_{k}=arg\underset{X}{max}X^{\prime }S_{k}X,subjectto∥X∥=1.$$

This part of the algorithm is carried out for all microstates.

The algorithm alternates iteratively between groups of Equations (7)–(9) and Equations (10) and (11). The process is started with an initial guess for either the microstates $\Gamma_{k}$ or the labels $L_{t}$, and terminated when the functional *F* differs negligibly, in general, $F_{min}<10^{-6}$. Thus, the minimum of function is given by

(12) $$F_{min}={\hat{\sigma }}_{\mu }^{2}=\sum_{t=1}^{N_{T}}\left(V_{t}^{\prime }\cdot V_{t}-{\left(\Gamma_{k}^{\prime }\cdot V_{t}\right)}^{2}\right)/\left(N_{T}\left(N_{s}-1\right)\right).$$

After the alternating iteration of the microstate algorithm, the K-means algorithm is used to cluster multichannel EEG data *X*. Then, $\Gamma_{k}\;\left(k=1,2,\cdots ,N_{\mu }\right)$ is taken as the cluster center, and the distance between each point to the cluster center is calculated. Each point is clustered into the class whose center is the nearest, i.e.,

(13) $$C_{t}=arg\underset{k}{min}{∥V_{t}-\Gamma_{k}∥}^{2},$$

where $C_{t}$ is the class of the point. Then, the average of all the points in each cluster is calculated, and the average is regarded as the new cluster center $m_{k}$,

(14) $$m_{k}=\frac{1}{N}\sum_{t|c_{t}=k}V_{t},$$

where *N* is the number of points in the class *k*.

Thus, it is iterated repeatedly between Equations (13) and (14) until the end. Finally, the topographical map is built according to the final cluster center $m_{k}$.

An algorithm is developed for estimating the microstates, based on a modified version of the classical K-means clustering method, in which cluster orientations are estimated. Consequently, each instantaneous multichannel evoked potential measurement is classified as belonging to some microstates, thus producing a natural segmentation of brain activity.

#### 2.3.3. GFP

Lehman and Skrandies proposed the method of calculating the Global Field Power (GFP) in 1980 and 1982 respectively [15,38], which allowed quantification of the integrated electrical activity of the topographical map by calculating spatial standard deviations.

$V_{t}={\left[v_{1}^{t},v_{2}^{t},\cdots ,v_{N_{s}}^{t}\right]}^{T}$ is a $N_{s}\times 1$ vector consisting of the scalp electric potential measurements at time instant $t\;(t=1,\cdots ,N_{T})$, the function for calculating the GFP is as follows:

(15) $$v_{t}^{\prime }=\frac{1}{N_{s}}\sum_{i=1}^{N_{s}}{\left(v_{i}^{t}\right)}^{2},$$

where $v_{t}^{\prime }$ is the value of GFP at time instant *t*, and $N_{s}$ is the number of channels to record EEG data.

#### 2.3.4. The Lateralization Analysis

After the microstate algorithm, the $\Gamma_{k}\;\left(k=1,2,\cdots ,N_{\mu }\right)$ is used to calculate the activation of the parietal occipital area in the mode of microstate. The D-value between the brain activities in right parietal area occipital and left parietal occipital area for each subject at the 12 visual stimulations is given by

(16) $$Z_{HZMX}=\sum_{i=1}^{N}x_{n}-\sum_{j=1}^{N}y_{n},$$

where $Z_{HZMX}$ is the D-value, $x_{n}\epsilon Left\;area,\;y_{n}\epsilon Right\;area,x_{n}\epsilon \Gamma_{k}\;y_{n}\epsilon \Gamma_{k}$. In Equation (16), $x_{n}$ and $y_{n}$ are the one-to-one correspondence between the left and right hemispheres. For example, $x_{n}$ is the channel of O1, correspondingly, $y_{n}$ is the channel of O2. Then, the mean and standard deviation of the $Z_{HZMX}$ for the all subjects at the same stimulus type are calculated.

Furthermore, according to the algorithm of the lateralization analysis, giving the N=3, the channels of O1, P3, P7, O2, P4 and P8 were chosen to study the lateralization of the microstates mode A.

## 3. Results

### 3.1. Feature of Microstates in Mental Rotation

**Figure 2:**
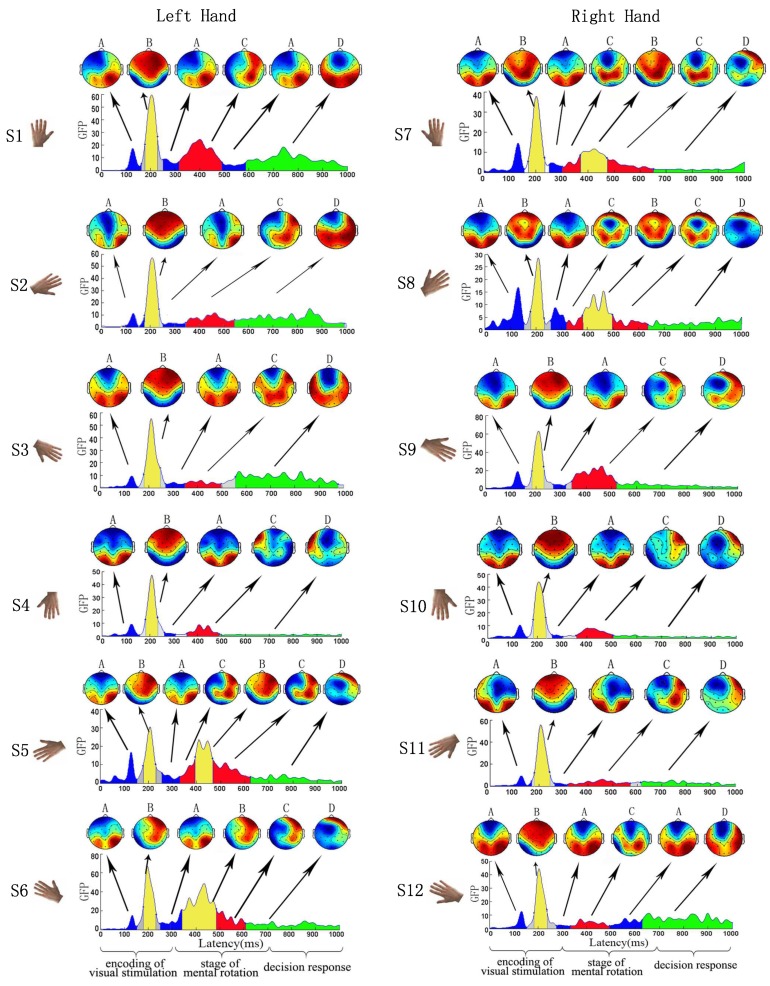
Feature of microstate modes (i.e., A, B, C, D) for 12 visual stimuli. Global field power (GFP) and functional microstates of mental rotation were analyzed by 12 visual stimuli. Four functional microstate modes (marked in blue, yellow, red and green, respectively) were found in the stimuli S1 to S12. The topographical maps were shown to be located at the time intervals corresponding to the microstates. During the encoding of visual stimulation, the microstates sequence had a specified sequential mode A→B→A in all different visual stimuli.

In this work, the EEG data of 11 subjects was averaged at the same stimulus type. Mental rotation task was dissected into four microstate modes (i.e., A, B, C, D) in all stages, which were observed in 12 different visual stimuli S1 to S12. The optimal number of microstates, estimated using a widely accepted cross-validation criterion [13,29] was 4 for 12 visual different visual stimuli, respectively. Then, the microstates were represented by four topographical maps. We observed the following spatial-temporal patterns from Figure 2: (1) from the topographical map of microstates, the microstates mode A has an active region in the bilateral parietal occipital area; the microstates mode B has the active region in the bilateral anterior hemisphere (frontal and central cerebral lobe); the microstates mode C has an active region in the parietal lobe and frontal lobe; the microstates mode D has an active region in the frontal lobe; (2) with all types of stimuli during the encoding of visual stimulation, the microstates sequence inherited the periodicity form EEG, i.e., A→B→A. Specifically, the activation area in the brain first appeared in the bilateral parietal occipital area and subsequently moved to the bilateral anterior hemisphere (frontal and central cerebral lobe), and then went back to the bilateral parietal occipital area; (3) mostly, microstates mode A (in blue in GFP panels, Figure 2) and microstates mode B (in yellow in GFP panels, Figure 2) were corresponding to the encoding of visual stimulation. Microstates mode C (in red in GFP panels, Figure 2) was corresponding to the stage of mental rotation. Microstates mode D (in green in GFP panels, Figure 2) was corresponding to the stage of decision response. Subsequently, we paid close attention to the feature of microstates in the encoding of visual stimulation.

The mean duration of microstate modes and the percentage of time occupation (“coverage”) of microstate modes were computed for the four microstate modes in all individuals. We assessed these measures of microstate modes during the encoding of visual stimulation. From Table 2, only considering the microstates modes A and B, the mean duration of microstate varied between 63.11 ms and 110.23 ms, and microstate coverage ranged between 21.15% and 36.93%. Furthermore, across each subject and each stimulus type, percent of total time covered by the microstates modes A and B was 79.44% (standard deviation (SD): 18.43%). Then, across each subject, the four microstate modes accounted for 86.2% (SD: 7.3%) of the data variance during the encoding of visual stimulation. Furthermore, we counted the number of the generalized microstate sequence A→B→A. Note that here the generalized microstate sequence is defined to include the microstate sequences A→B→A (31.06%), D→A→B→A (14.39%), C→A→B→A (14.39%), A→C→B→A (5.30%), and so on, which with A→B→A sequence embedded. The mean proportion of generalized microstates sequences A→B→A was 76.52% (SD: 10.59%), which verified this specified mode during encoding of visual stimulation. On the other hand, when the EEG data of 11 subjects was averaged at the same stimulus type, the microstates sequence had the underlying EEG periodicity A→B→A in all different visual stimuli. Thus, it demonstrates that individuals had a generalized microstate sequence A→B→A to a large extent on all subjects with all subjects.

### 3.2. The Relationship between Microstate and ERP

**Figure 3:**
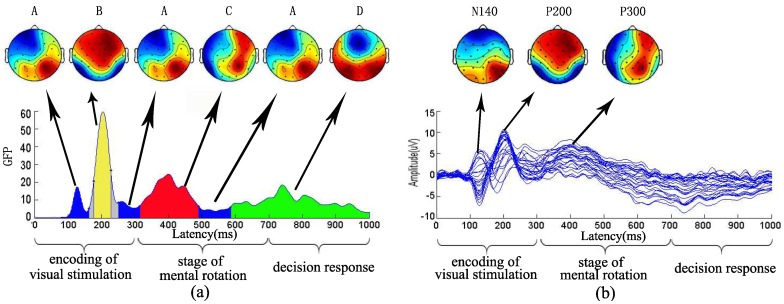
The relationship between microstates and ERP. (**a**) the microstate modes of S1 visual stimulation; (**b**) grand-averaged ERP curves at 28 electrodes for S1 visual stimulation and three brain topographical maps of ERP located at the time intervals corresponding to the N140, P200, P300 components, respectively. In the brain topographical maps, the deep red color represents higher activation and the deep blue color represents weaker activation.

Meanwhile, we examined the relationship between the topographical map of microstates and brain map of ERP in S1 visual stimulation in Figure 3. Microstate analysis was to describe the dynamics of brain function from a time sequence of Figure 3a. In contrast to the ERP results, for 12 visual stimuli, grand-averaged ERP curves at 28 electrodes were calculated with the same stimulus type for 11 subjects by averaging. As can be seen from Figure 3b, brain mappings of N140, P200, P300 were illustrated. Brain areas in the bilateral parietal occipital area were observed with prominent activation of N140. Significant P200 activation was found in anterior cerebral hemisphere. In addition, the P300 component in 400 ms was found in the frontal and parietal lobe. Moreover, from the Figure 3, the brain activation area of the first microstates mode A, microstates modes B and C showed smaller topographic differences with the topographical maps of N140, P200 and P300 component, respectively. Differently, ERP analysis only examined the components with an obvious peak value in Figure 3b but could not show the component at about 300 ms. However, according to the microstates analysis, there was a second microstates mode A at about 300 ms between the microstates mode B and the microstates mode C.

### 3.3. Hemispheric Lateralization of the Microstates Mode A

**Figure 4:**
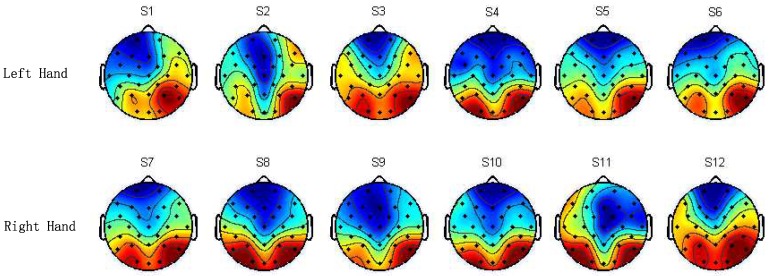
Grand average of microstates topographical maps of the mode A among 11 subjects for the 12 different visual stimuli. In the topographical maps of microstates, the deep red color represents a stronger brain activity and the deep blue color represents weaker brain activity. Note that microstates mode A showed a clear hemispheric lateralization at the parietal occipital area.

The microstate topographical maps of the mode A of 12 visual stimulations demonstrated a clear hemispheric lateralization, and the activation of the right parietal occipital area was stronger than that of the left parietal occipital area (Figure 4).

In this work, the mean of O1, P3 and P7 was taken as the brain activity of left parietal occipital area and the mean of O2, P4 and P8 was taken as the brain activity of the right parietal occipital area. Table 3 showed the brain activities with the corresponding mean and standard deviation with the all subjects. These measures were subjected to a paired *t*-test for repeated measurements. The *t*-test showed that all stimulations, except for S3, S8 and S12 induced significantly increased brain activities in the right area compared to the left counterpart area (p<0.05) Table 3.

### 3.4. “Angle Effect" on the Duration of the Second Microstates Mode A

**Figure 5:**
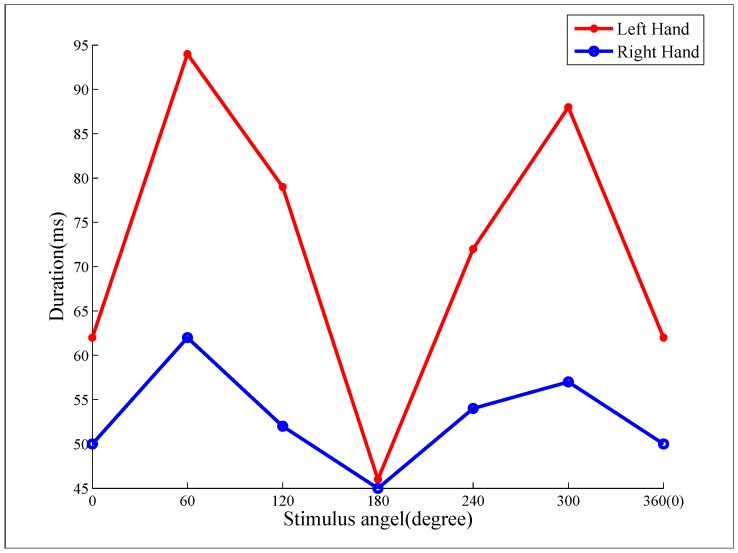
The duration of the second microstates mode A. The duration had a significant “angle effect”: shorter duration in cases of $0^{\circ }$ (S1, S7) and $180^{\circ }$ (S4, S10) for both the left- and the right-hand stimuli, and longer duration in the case of stimuli with left-hand pictures compared with that of stimuli with right-hand pictures at the same rotated angle.

In order to understand the details of the cognitive process in mental rotation with microstate analysis, the grand-averaged duration of second microstates mode A at visual stimuli for 11 normal subjects was shown in regard to angle factor in left and right hands, respectively. The duration demonstrated a significant “angle effect” (Figure 5): (i) there was longer duration for the left-hand stimuli than the right-hand stimuli in all six different angles; (ii) there was tendency that the duration was shorter for the cases of vertical stimuli (i.e., $0^{\circ }$ and $180^{\circ }$), but longer for the cases of stimuli with angles apart from vertical direction (i.e., $60^{\circ }$, $120^{\circ }$, $240^{\circ }$ and $300^{\circ }$) for both the left- and right-hand stimuli. Generally, the duration was a minimum when the stimuli with upright (S1, S7) or inverted upright (S4, S10) hand pictures for both left and right hands.

## 4. Discussion

In this paper, we analyzed the cognitive process of mental rotation based on EEG microstates for 11 subjects. We only made the statistical analysis within a group using a paired *t*-test. The statistical analysis within the group did not require a high number of subjects. The results can be summarized as follows: firstly, the microstates sequence displayed periodic information and the activation area showed the specific alternate activation process. Secondly, the topographical of microstates and brain map of ERP revealed the differences and similarities between the ERP and microstates. Thirdly, the microstates mode A showed a hemispheric lateralization for all 12 of the visual stimuli. Fourthly, in addition, a shorter duration of the second microstates mode A displayed an “angle effect” in cases of stimuli with vertical hand pictures.

### 4.1. Alternate Activation Process of the Brain Activation Area

The microstates topographical map indicated that the activation area had an alternate activation process: strong brain activity first appeared in the bilateral parietal occipital area and then moved to the anterior cerebral hemisphere (frontal and central cerebral lobe) and subsequently went back to the bilateral parietal occipital area (Figure 2). Encoding of visual stimulation, an exogenous response to the external visual stimulus will encode various physical attributes including shape, color, and spatial orientation of the visual stimulus image [32]. The parietal lobe is primarily used to encode spatial information, which will be activated when the visual stimulus is spatially encoded [32]. Since the visual stimulus images were used in the experiment with left- and right-hand images rotated to different angles, the motor cortex which was associated with the hand would be activated. Therefore, the frontal and central cerebral lobe would be activated during the encoding of visual stimulation. Furthermore, the stage of mental rotation is a kind of implicit spatial rotation of the visual stimulus, which is similar to the real rotation of the visual stimulus to the correct spatial position, requiring the participation of parietal lobe. Decision response is the decision stage of the visual rotation stimulus, which requires the frontal lobe to participate and the frontal lobe is responsible for decision-marking. At least some possible phenomena could explain this observation. Firstly, some studies of mental rotation tasks with hand stimuli demonstrated significant motor area activations, which indicated the important role of the motor cortex during the mental rotation cognition [39]. Furthermore, neuroimaging studies with event-related functional magnetic resonance imaging (fMRI) revealed that a mental rotation task could activate dorsolateral promoter cortex in the early cognitive encoding of visual stimulation, which confirmed the direct involvement of the motor-related areas activation in encoding of visual stimulation [40].

Furthermore, it was interesting that the microstates sequence had a specific sequential mode A→B→A. However, this kind of sequential mode of brain activity in mental rotation has not been reported yet in previous studies. Yan et al. used the distribution of P200 components of ERPs to reveal the activation area during the encoding of visual stimulation, and reported that the activation area mainly appeared in the bilateral hemisphere, including the prefrontal cortex, frontal lobe, central cerebral lobe and the right parietal lobe. The activation area of P200 components was likely to correspond to the microstates mode B observed in our study [32]. Chen et al. explored the change of the ERP brain topographical map of mental rotation in schizophrenia. They found that brain activation was primarily distributed in the frontal, temporal and occipital lobe during 0–200 ms. The duration of 0–200 ms mainly corresponded to the processing of visual stimulus, searching visual and recognizing objects. Then, the stronger brain activity was mainly distributed in the parietal occipital area during 200–300 ms. Therefore, the activation area in brain shifted from the anterior cerebral hemisphere to the bilateral parietal occipital area corresponded to our microstate sequential mode B→A [41]. However, no ERP study on mental rotation has ever reported mode shifting like A→B→A. The microstates sequence adds the unique feature of reflecting the underlying EEG periodicity. Thus, we provide evidence that periodicity is an intrinsic feature of EEG derived microstates sequence. These results suggest that microstate analysis, which considers the spatial-temporal information of brain activity, may provide new insights on mental rotation than traditional ERP analysis.

### 4.2. Microstate and ERP in Mental Rotation

Similarly to what was observed in the microstates topographical map and ERP topographical map, the microstates modes A, B, and C were corresponded to N140, P200 and P300. Furthermore, the study demonstrates that two differences can be detected in topographical map of microstates and brain map of ERP. Firstly, according to ERP analysis, some details of the encoding of visual stimulation could not be found. From the experimental results (Figure 3), during the encoding of visual stimulation, the brain had an alternation activation process of the brain activation area, i.e., A→B→A. In contrast to the ERP results (Figure 3b) only found the N140, P200, P300 components, and then only examined the components with a distinct peak value. Furthermore, microstates analysis examines the changes in the brain topographical maps from the perspective of the whole brain pattern over space and had the stage representation ability from the whole process in mental rotation. Differently, ERP analysis could not show the any component at the stage of decision response (Figure 3b). Because there was no significant peak value at the decision stage, the microstate analysis can study spatial-temporal dynamics of brain.

### 4.3. Hemispheric Lateralization for Activation Area

The microstates mode A had a hemispheric lateralization and displayed a significantly stronger right-hemispheric activity in the right parietal occipital compared to left counterpart region (Figure 4). Windischberger and Jordan et al. used the functional magnetic resonance imaging (fMRI) to study mental rotation and found that the parietal cortex would be activated during the stage of visual stimulation encoding in mental rotation regardless of the stimulus pictures that were two-dimensional or three-dimensional. Then, activation of the right parietal lobe was stronger than that on the left parietal lobe [42,43]. Muthukumaraswamy et al. studied the ERP topographical map and reported that the right parietal lobe of the mental rotation process was activated [44]. Thus, there was hypothesized that the right posterior parietal lobe might be involved in fundamental low-level attentional processes that “act as the lowest common denominator for many types of cognitive processes”. However, in spatial and nonspatial attentional conditions, the posterior parietal lobe was activated. It was recruited more for spatial tasks than for nonspatial ones. This suggested that, apart from its role in attention, there might be some functional specialization for spatial processing in the right parietal cortex. Therefore, our results were in line with those reported in previous studies.

### 4.4. “Angle Effect" on Duration of the Second Microstates Mode A

A significant difference of the second microstates mode A between the vertical direction and dip direction of hand pictures was observed for all six different angles (Figure 5). In particular, the duration of the second microstates mode A was shorter for the stimuli with hand pictures of vertical direction (i.e., $0^{\circ }$ in S1 and S7, and $180^{\circ }$ in S4 and S10) compared with the stimuli with hand pictures rotated to other angles. However, most studies showed that, with the increase of the rotation angle of the visual stimulus picture from the upright position ($0^{\circ }$) to $180^{\circ }$, the response time gradually increased and reached the maximum at $180^{\circ }$ in normal subjects. These results suggested that more processing time was required when subjects processed the stimuli rotated to larger angles apart from upright position (i.e., $0^{\circ }$) [32,45,46]. However, interestingly, during the encoding of visual stimulation, the vertical direction of hands might show a faster speed of coding processing compared to sloping angles. The traditional ERP research does not measure duration of cognitive processing and requires a combination of behavioral response time. However, the microstate analysis can measure the duration during the stage of the cognitive processing. Therefore, the microstate analysis provides new insights for mental rotation.

## 5. Conclusions

In summary, we investigated the cognitive process of mental rotation during the encoding of visual stimulation using the microstates method. The microstates sequence A→B→A inherited periodicity from the underlying EEG dynamics during encoding of visual stimulation, that is, the activation area had a specific alternate activation process. The topographical map of microstates mode A showed the hemispheric lateralization due to a stronger activation appearing in the right parietal occipital. It was quite remarkable that four (and even fewer) topographical maps captured such important EEG features as alternate activation process and hemispheric lateralization. Furthermore, the “angle effect” in duration of the second microstates mode A was highly significant. These results might provide new insights into understanding of the cognitive process during mental rotation. Cognitive tasks can inspire advanced cognitive functions related to movement. This is the foundation of brain cognition research. Furthermore, in order to reveal the impact of stroke lesions focusing on the cognitive mechanism, it is possible to combine the symptoms and locations of areas with impaired motor function in stroke patients. Subsequently, the study of cognitive activity mechanism can be applied to the research of brain diseases and artificial brain intelligence.

## Figures and Tables

**Table 1:** Stimulus types.

Left Hand				Right Hand		
Name	Rotation Angle	Probability		Name	Rotation Angle	Probability
S1	$0^{\circ }$ 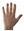	25%		S7	$0^{\circ }$ 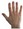	25%
S2	$60^{\circ }$ 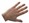	12.5%		S8	$60^{\circ }$ 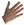	12.5%
S3	$120^{\circ }$ 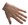	12.5%		S9	$120^{\circ }$ 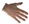	12.5%
S4	$180^{\circ }$ 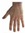	25%		S10	$180^{\circ }$ 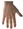	25%
S5	$240^{\circ }$ 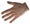	12.5%		S11	$240^{\circ }$ 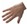	12.5%
S6	$300^{\circ }$ 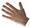	12.5%		S12	$300^{\circ }$ 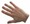	12.5%

**Table 2:** The duration and coverage of microstate mode (Mean and SD) in 11 subjects and 12 visual stimuli.

Parameters	*A*		*B*		*A*		*A* + *B* + *A*	
	Mean	SD	Mean	SD	Mean	SD	Mean	SD
Duration (ms)	110.23	54.44	70.33	29.01	63.11	35.29	237.45	61.77
Coverage (%)	36.93	17.96	23.38	8.61	21.15	11.28	79.44	18.43

**Table 3:** Grand average and SD of brain activities in microstates mode A among all subjects for visual stimuli S1 to S12. A paired *t*-test showed a significant difference between the brain activities in the right parietal occipital area and the left counterpart area.

Hand	Stimulus Type	Left Area			Right Area		*p*-Value
		Mean	SD		Mean	SD	
Left Hand	S1	1.43	2.30		2.66	3.31	0.021
	S2	1.03	1.11		1.53	1.18	0.045
	S3	1.38	2.22		1.80	2.28	0.161
	S4	0.76	1.44		1.15	1.52	0.021
	S5	−0.10	2.12		0.87	1.62	0.026
	S6	1.35	2.15		3.05	3.12	0.010
Right Hand	S7	1.39	2.15		2.50	3.01	0.047
	S8	1.13	2.30		1.94	1.96	0.1125
	S9	0.03	2.35		1.36	1.83	0.020
	S10	0.58	1.38		1.74	1.84	0.012
	S11	0.27	1.85		1.64	1.84	0.021
	S12	0.43	1.68		1.37	1.91	0.089
